# An Integrated Systems Pharmacology Approach Combining Bioinformatics, Untargeted Metabolomics and Molecular Dynamics to Unveil the Anti-Aging Mechanisms of *Tephroseris flammea*

**DOI:** 10.3390/biom15121740

**Published:** 2025-12-15

**Authors:** Min Hyung Cho, Haiyan Jin, JangHo Ha, SungJune Chu, SoHee An

**Affiliations:** 1 Bioinformatics and Molecular Design Research Center (BMDRC), Songdogwahak-ro 85, Yeonsu-gu, Incheon 21983, Republic of Korea; 2 Department of Integrative Biotechnology, Yonsei University, Songdogwahak-ro 85, Yeonsu-gu, Incheon 21983, Republic of Korea

**Keywords:** *Tephroseris flammea*, skin anti-aging, network pharmacology, untargeted metabolomics, molecular dynamics, UV-induced photoaging

## Abstract

Skin aging, driven by oxidative stress, UV exposure, inflammation, and extracellular matrix degradation, necessitates the discovery of safer, multi-target natural products. We established an integrated pipeline combining UHPLC–MS/MS metabolomics, computational methods (network pharmacology, molecular docking, and dynamics simulation), and in vitro bioassays to efficiently discover and mechanistically characterize anti-aging compounds from novel botanical sources. We applied this pipeline to identify and evaluate Tephroseris flammea, a previously unassessed plant. Metabolomic profiling identified 21 compounds, including flavonoids, phenylpropanoids, and pyrrolizidine alkaloids. These compounds were linked via network pharmacology to 226 skin-aging-related targets, primarily involving inflammation (via AKT1, RELA) and matrix degradation (via MAPK3). Molecular docking and 100 ns molecular dynamics simulations confirmed stable ligand-target interactions with favorable binding energies. Validating these in silico predictions, the *T. flammea* extract demonstrated significant antioxidant activity and effectively suppressed key inflammatory mediators (IL-6, TNF-α, COX-2) and MMP-1 levels in UVB-exposed fibroblasts, notably without significant cytotoxicity. Collectively, this study validates the utility of our pipeline to mechanistically characterize complex botanicals, revealing that *T. flammea* contains multifunctional compounds modulating critical inflammatory and matrix-regulatory cascades. This work validates our pipeline for identifying novel, mechanistically defined ingredients from complex botanical sources.

## 1. Introduction

Skin aging is a complex, multifactorial process influenced by both extrinsic and intrinsic determinants that progressively compromise the structural integrity and physiological function of the skin [1]. Extrinsic aging is predominantly driven by chronic ultraviolet (UV) exposure and oxidative stress provoked by environmental pollutants, infrared radiation, and lifestyle-associated factors [2]. Conversely, intrinsic aging originates from genetic programming and metabolic decline, resulting in cellular senescence, mitochondrial inefficiency, and a gradual disruption of extracellular matrix (ECM) homeostasis [3,4,5]. These insults activate epidermal growth factor- and cytokine-mediated kinase cascades—notably the mitogen-activated protein kinase (MAPK) and phosphatidylinositol-3-kinase (PI3K)/Akt pathways. This activation in turn enhances the phosphorylation of pro-inflammatory transcription factors such as nuclear factor-kappa B (NF-κB) and activator protein-1 (AP-1), driving the upregulation of downstream enzymes including matrix metalloproteinases (MMPs) and elastases. The sustained activation of such oxidative and inflammatory pathways accelerates collagen degradation, elastin fragmentation, and ECM remodeling, ultimately producing visible manifestations including loss of elasticity, coarse texture, uneven pigmentation, and wrinkle formation [6,7,8]. Furthermore, persistent stimulation of these pathways induces chronic oxidative injury, immunosuppression, and premature apoptosis of dermal cells, establishing a self-perpetuating cycle of inflammation and ECM breakdown [9,10]. Clinically, these molecular perturbations give rise to dryness, epidermal thickening, pigmentation disorders, and actinic wrinkling and, in severe cases, may contribute to photocarcinogenesis [9,11,12]. Beyond esthetic relevance, the cumulative biological and psychological burden of skin aging emphasizes the necessity for preventive and therapeutic strategies that specifically target oxidative and inflammatory cascades at the molecular level [13].

Despite considerable advances in dermatological and cosmetic research, most current interventions afford only limited protection against the multifactorial nature of skin aging [14,15,16]. This has intensified the demand for safe, multifunctional agents capable of simultaneously modulating the oxidative and inflammatory cascades underlying dermal degeneration. Natural products (NPs) constitute one of the most abundant reservoirs of pharmacologically active molecules and have consistently served as templates for modern drug discovery [17,18]. Through evolutionary refinement within biological systems, these metabolites have acquired high scaffold diversity, stereochemical complexity, and advantageous physicochemical traits—such as increased sp^3^ character, rigidity, and hydrophilicity—that facilitate selective modulation of protein–protein interactions and intracellular signaling networks [19]. Consequently, NPs occupy a unique and biologically relevant chemical space that reflects natural adaptation to oxidative and inflammatory stress [20]. This intrinsic compatibility endows many phytochemicals with the capacity to act as senotherapeutic or cytoprotective agents, attenuating cellular senescence, restoring mitochondrial redox balance, and reactivating endogenous repair pathways. Exemplary compounds, including fisetin, quercetin, resveratrol and sulforaphane, have been shown to eliminate senescent cells or activate the KEAP1–NRF2 antioxidant response, thereby maintaining proteostasis and cellular resilience [21,22,23,24]. Compared with synthetic analogs, such multitarget natural metabolites display lower cytotoxicity, superior biocompatibility, and synergistic regulation of redox, inflammatory, and extracellular matrix pathways [25,26]. Collectively, natural products represent an evolutionarily validated and mechanistically versatile source for discovering safe and multifunctional anti-aging candidates.

Most previous investigations on natural products have relied on rather traditional methods, such as bioassay-guided fractionation or target-specific screening. While such reductionist strategies are valuable for validating known species or previously characterized compounds, they are inherently limited in identifying entirely new natural product candidates from fragmentary or low-signal information—data that often remains insufficient to influence later intellectual property claims [27]. Moreover, these approaches depend heavily on the expertise and intuition of individual researchers, thereby constituting a major bottleneck for systematic or large-scale discovery efforts. Recent advances in computational and omics-based technologies have begun to mitigate these limitations. Expanding global compendia of chemical, biological, and clinical data generated by the scientific community now provide an unprecedented reservoir of “known” entities that, when accessed through systematic informatics pipelines, can serve as an effective foundation for data-driven exploration [28,29]. Network pharmacology, in conjunction with molecular docking and molecular dynamics simulations, enables systems-level interpretation of multi-component and multi-target interactions, whereas metabolomics allows the comprehensive chemical profiling of entire phytochemical repertoires [30]. The integration of these databases and analytical tools establishes an information-based framework that links molecular identity to biological function, facilitating rational prioritization of promising species prior to extensive experimental validation [29,31,32]. Such an approach not only accelerates the discovery of novel botanical resources but also refines mechanistic insight by correlating compound classes with specific signaling pathways—for example, those involved in oxidative and inflammatory processes underlying skin aging.

**Figure 1:**
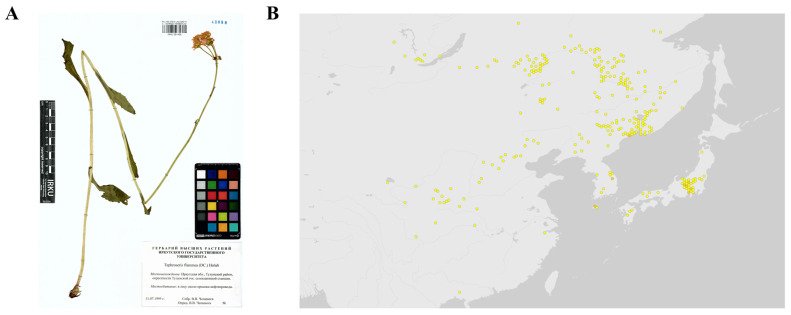
Botanical characterization of *T. flammea*. (**A**) Preserved specimen image of *T. flammea*. Sourced from GBIF (https://www.gbif.org/occurrence/3908987869, accessed on 7 November 2025). (**B**) Native distribution of *T. flammea*. Retrieved from GBIF Georeferenced records. Yellow dots: georeferenced point records representing individual occurrences.

Within this framework, we investigated plant species native to the Korean Peninsula and neighboring East Asian regions to identify phytochemicals with potential anti-aging effects. Among them, *Tephroseris flammea* (Turcz. ex DC.) Holub. (Figure 1) emerged as a particularly promising candidate. The botanical morphology of this species is illustrated in Figure 1A, while its geographical range is mapped in Figure 1B. *T. flammea* is an East Asian perennial herb that inhabits subalpine and temperate grasslands, distributed sporadically across the Korean Peninsula, northeastern China, and the Japanese archipelago [33]. Although morphologically allied to *T. kirilowii* and other Eurasian congeners, *T. flammea* represents a phylogenetically distinct lineage that has not been chemically or pharmacologically characterized. Members of the *Tephroseris* genus are known to biosynthesize structurally diverse secondary metabolites, including flavonoids, phenolic acids, and pyrrolizidine or sesquiterpene alkaloids—classes that frequently exhibit antioxidant, anti-inflammatory, and cytoprotective activities in related taxa [34]. However, no systematic study has yet elucidated the metabolomic composition, bioactive constituents, or mechanistic relevance of *T. flammea* to skin aging. This lack of characterization contrasts sharply with the growing pharmacognostic and cosmeceutical interest in Asteraceae plants, underscoring the need for comprehensive profiling of this underexplored species.

To address this knowledge gap, the primary objective of this study was to systematically elucidate the bioactive landscape and mechanistic targets of *T. flammea* relevant to skin aging. The study employed a multi-layered analytical pipeline that combines untargeted UHPLC–MS/MS metabolomics, network pharmacology, molecular docking, and molecular dynamics simulations, coupled with in vitro bioassays in UVB-stimulated fibroblast models. This top-down strategy was designed to link chemical identity with biological function prior to isolation or fractionation, thereby accelerating evaluation of complex plant matrices. Specifically, our objectives were to (i) characterize the comprehensive metabolite profile of *T. flammea*, (ii) predict and validate its putative molecular targets associated with oxidative and inflammatory skin aging, and (iii) experimentally verify the predicted effects through quantitative bioassays. Collectively, this study provides the first integrative biochemical and mechanistic insight into *T. flammea* and exemplifies the applicability of our informatics-integrated discovery platform to the rational exploration of uncharacterized botanical resources.

## 2. Materials and Methods

### 2.1. Preparation of Tephroseris flammea (Turcz. ex DC.) Holub. Whole Plant Extract

**Figure 2:**
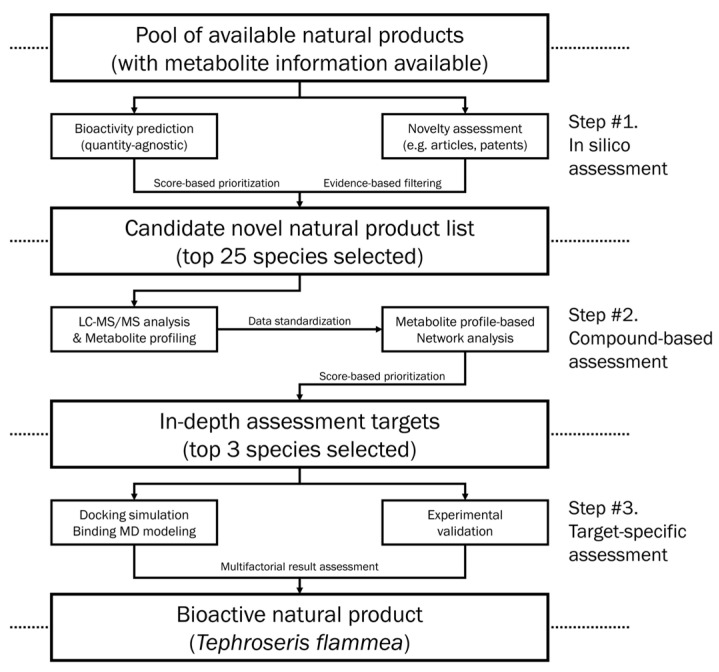
Workflow diagram for identification of *T. flammea* as a novel active natural product.

The plant extract (KPM031-051, code no: PMKR0045) used in this research was obtained from the Natural Product Central Bank at the Korea Research Institute of Bioscience and Biotechnology (Cheongju, Republic of Korea) (Figure 2). A voucher specimen (KRIB 0014018) is kept in the herbarium of the Korea Research Institute of Bioscience and Biotechnology. The plant was collected from Taebaek-si, Gangwon-do, Korea in 2007. Immediately after collection, the material was dried in the shade to deactivate enzymatic processes and eliminate moisture stored in a controlled environment at −4 °C in the dark to ensure long-term metabolite stability. The plant (53 g) dried in the shade and powdered was added to 1 L of methanol 99.9% (HPLC grade) and extracted through 30 cycles (40 kHz, 1500 W, 15 min ultrasonication–120 min standing per cycle) at room temperature using an ultrasonic extractor (SDN-900H, SD-ULTRASONIC Co., Ltd., Seoul, Republic of Korea). After filtering (Qualitative Filter No. 100, HYUNDAI MICRO Co., Ltd., Seoul, Republic of Korea) and drying under reduced pressure, *T. flammea* extract (2.56 g) was obtained.

### 2.2. UHPLC–MS/MS Analysis and Metabolite Identification

#### 2.2.1. UHPLC–MS/MS Analysis of *T. flammea* Extract

To perform qualitative analysis of the major compounds in the *T. flammea* extract, the sample was dissolved in methanol at a concentration of 1 mg/mL and filtered through a 0.2 μm PTFE syringe filter. UHPLC-MS/MS analysis was subsequently performed using a system combining an Ultra High Performance Liquid Chromatography (Vanquish Horizon UHPLC, Thermo Fisher Scientific, Waltham, MA, USA) with an Orbitrap ID-X Tribrid mass spectrometer (Thermo Fisher Scientific, Waltham, MA, USA). A sample injection volume of 1 μL was separated using a Hypersil GOLD™ Vanquish column (150 × 2.1 mm, 1.9 μm; Thermo Fisher Scientific, Waltham, MA, USA) maintained at a column oven temperature of 40 °C. The mobile phase consisted of Solvent A (Water containing 0.1% formic acid) and Solvent B (Acetonitrile containing 0.1% formic acid). Elution was performed at a flow rate of 0.3 mL/min using the following linear gradient: 0–3 min (5.0% B), 3–7 min (5.0–11.5% B), 7–8 min (11.5–12.0% B), 8–15 min (12.0–14.0% B), 15–25 min (14.0–25.0% B), 25–27 min (25.0–50.0% B), 27–29 min (50.0% B), 29–34 min (50.0–60.0% B), 34–36 min (60.0–100.0% B), 36–50 min (100.0% B), 50–55 min (100.0–5.0% B), and 55–60 min (5.0% B). MS analysis utilized a Heated Electrospray Ionization (H-ESI) source. The spray voltage was set to 3500 V in positive mode and 2500 V in negative mode. The flow rates for the Sheath gas, Aux gas, and Sweep gas were maintained at 50, 10, and 1 (arbitrary units), respectively. The ion transfer tube temperature was 320 °C, and the vaporizer temperature was set to 300 °C. Full MS scans were performed in the Orbitrap analyzer at a high resolution of 120,000, covering the mass range of *m*/*z* 100–1500, with the RF lens set to 35%. MS/MS scans were acquired in data-dependent acquisition (DDA) mode at a resolution of 15,000. Fragmentation was induced using assisted Higher-energy Collisional Dissociation (HCD) with collision energies set to 30%, 40%, and 50%.

#### 2.2.2. Metabolite Identification

The raw data files were imported into MS-DIAL (v5.5.241113), an open source software platform (https://systemsomicslab.github.io/compms/msdial/main.html) (accessed on 7 January 2025), for peak picking, deconvolution, deisotoping, alignment, and formula prediction. Metabolites were characterized based on their exact monoisotopic mass, retention time (t_m_), and MS/MS fragmentation patterns. Metabolite identification was performed by comparison with online chemical DBs (e.g., PubChem) and validated against reference literature, ensuring an acceptable confidence level (Level 2a: probable structure based on MS, MS/MS, and library/bibliography search) [35].

### 2.3. Reference-Based Identification of Potential Targets for Skin Aging

To define a reference set of molecular targets associated with skin aging, the term “skin aging” was used as a search query in the GeneCards and Open Targets Platform databases. Each database was queried independently to retrieve gene entries annotated with disease relevance. The resulting target lists were subsequently filtered according to the gene–disease association score (Genecards relevance score ≥ 0.2, Open Targets association score ≥ 0.001), removing possible noises. To further improve the coverage of senescence-related factors, the manually curated SenSkin™ database was additionally incorporated, providing experimentally validated genes linked to cutaneous aging and cellular senescence [36]. All retrieved entries were standardized according to their official gene symbols and merged to remove redundancy, resulting in a unified non-redundant target list for downstream network pharmacology and pathway analyses.

### 2.4. Metabolite Screening and Compound–Protein Interaction (CPI) Partner Prediction

Secondary metabolites identified by UHPLC–MS/MS analysis were subjected to computational screening to predict their putative molecular targets. To eliminate background interference from ubiquitous small molecules, primary metabolites such as sugars, amino acids, fatty acids, and nucleotides were excluded, and only structurally defined secondary metabolites were retained for further analysis. The canonical SMILES representation of each compound was retrieved from PubChem to ensure interoperability across target prediction platforms.

Information on experimentally validated compound–protein associations was extracted from the PubChem bioassay database, which integrates curated interaction data from individual DBs including Comparative Toxicogenomics Database (CTD), Drug–Gene Interaction Database (DGIdb), and BindingDB, as well as primary literature-derived bioassay results (PubChem Bioassay). To complement these curated datasets, potential targets were also predicted de novo using SwissTargetPrediction and STITCH v5.0, restricting species selection to Homo sapiens. For SwissTargetPrediction, only interactions with a probability score ≥0.09 were retained. For STITCH, interactions with a combined confidence score ≥0.4 were considered medium-confidence, which allows to consider both experimentally verified and high-probability predicted interactions, allowing for a comprehensive network construction. Predicted and experimentally supported targets were merged, de-duplicated by UniProt accession number, and subsequently cross-referenced with the predefined skin aging–related gene set. The intersecting targets were defined as the bioactive target set of *T. flammea* for downstream network pharmacology and molecular docking analyses.

### 2.5. Network Pharmacology

#### 2.5.1. Protein–Protein Interaction Network Construction

To elucidate the pharmacological mechanisms underlying the anti–skin-aging effects of *T. flammea*, a compound–target–pathway interaction network was constructed. The validated and predicted compound–target pairs were imported into Cytoscape v3.10.4 to visualize the topological relationships between metabolites and their corresponding protein targets. The network was analyzed using the NetworkAnalyzer plugin to compute key topological parameters, including degree centrality, betweenness centrality, and closeness centrality, thereby identifying hub nodes with high regulatory significance.

The corresponding protein–protein interaction (PPI) network was generated using the STRING database v12.0, restricting the organism to Homo sapiens. Interactions with a confidence score ≥ 0.7 (high confidence) were retained, while disconnected nodes (‘singletons’) were removed. The resulting network was imported into Cytoscape v3.10.4 for visualization and further analysis.

#### 2.5.2. Pathway Enrichment Analysis

To characterize the biological functions of the core targets, Gene Ontology (GO) enrichment—including biological process (BP), cellular component (CC), and molecular function (MF) categories—and Kyoto Encyclopedia of Genes and Genomes (KEGG) pathway enrichment analyses were performed using the ShinyGO 0.85 platform. Enrichment significance was determined by a false discovery rate (FDR) <0.05 and gene count ≥ 3. Redundant or semantically overlapping GO terms were clustered using ShinyGO’s hierarchical grouping plot option. The top 15 GO terms and KEGG pathways were selected according to adjusted *p*-values and visualized consequently.

### 2.6. Molecular Dynamics

#### 2.6.1. Protein Structure Preparation

To perform molecular docking and dynamics analyses, protein structures corresponding to the top-ranked target genes—including both high-scoring protein–compound interaction candidates and hub genes identified from the *T. flammea* target network—were retrieved from the Protein Data Bank (PDB) (Supplementary Table S11). Preference was given to crystal structures complexed with small-molecule inhibitors or co-crystallized ligands whenever available (Table S12). Crystal structures were collected for sixteen target proteins, comprising STAT3, AKT1 (ATP-binding and allosteric sites), MMP9, TNF-α, PTGS2, EGFR (ATP-binding and allosteric sites), RELA, MAPK3, IL1B, MAPK14, AMPK, CASP3, GSK3B, PPARG, XDH, and NOS2. All utilized PDB structures are listed in Supplementary Table S11.

All protein structures were prepared using the Protein Preparation Wizard in Maestro (Schrödinger, LLC, New York, NY, USA, version 2025-1). Missing loops or side chains were modeled using Prime, and hydrogen atoms were added considering physiological pH (7.4). Protonation states of titratable residues were adjusted with PROPKA (pH 7.4), followed by optimization of hydrogen bonding networks.

Restrained energy minimization was carried out using the OPLS4 force field with a heavy-atom convergence threshold of 0.3 Å RMSD. Crystallographic water molecules located beyond 5 Å from the native ligand binding sites were removed, while those potentially involved in ligand stabilization were retained for subsequent docking calculations. All structural preparation settings were kept consistent across the 18 target complexes to ensure uniformity in downstream docking and simulation analyses.

#### 2.6.2. Molecular Docking

Molecular docking was conducted to generate binding poses using Glide (Maestro, Schrödinger LLC, New York, NY, USA, version 2025-1). The receptor grid generation module in Maestro was used to define the protein–ligand docking sites. Ligands were docked in standard precision (SP) mode against all target proteins.

For seventeen of the target structures, docking sites were defined based on the co-crystallized ligands present in the corresponding PDB complexes. In the case of RELA, which lacks a canonical small-molecule binding site and instead binds to DNA, two approaches were employed to determine a feasible binding pocket:

1. Selection of the region corresponding to the DNA-binding interface, and
2. Identification of potential druggable pockets using the SiteMap module in Maestro.

All docking settings were applied uniformly across the targets to ensure consistency in pose generation and scoring.

#### 2.6.3. MD Simulation

All-atom molecular dynamics (MD) simulations were performed using Desmond (Schrödinger LLC, New York, NY, USA, version 2025-1). Eight protein–ligand complexes—comprising Thesinine, Rutin, Kaempferol rutinoside, Chlorogenic acid, and Rivularine bound to AKT1 (allosteric site), MMP9, MAPK3, MAPK14, and XDH—were selected based on favorable docking scores and key residue interactions.

Each complex was solvated in an orthorhombic TIP3P water box with a 10 Å buffer distance from the solute boundary. Counterions (Na^+^ and Cl^−^) were introduced to neutralize system charge and maintain a physiological salt concentration of 0.15 M. The OPLS4 force field was applied for both protein and ligand parameterization. All MD simulations were conducted under NPT ensemble conditions (300 K, 1.01325 bar) using the Nosé–Hoover thermostat and Martyna–Tobias–Klein barostat. Initial equilibration followed the standard Desmond relaxation protocol [37].

For each of the eight complexes, three independent 100 ns simulation replicas were performed using distinct random seeds (2007, 4222, and 9177) to ensure reproducibility and statistically reliable sampling. Trajectory analyses were performed using the Simulation Interaction Diagram tool in Maestro. Protein backbone atoms were aligned to the initial frame prior to calculating Protein Cα RMSD to evaluate global protein conformational stability. Ligand RMSD was assessed to monitor ligand positional fluctuations within the binding pocket during the simulation.

RMSD values were calculated based on Equation (1):

(1) $${RMSD}_{X}=\sqrt{\frac{1}{N}\sum_{i=1}^{N}{({r^{\prime }}_{i}\left(t_{x}\right)-r_{i}(t_{ref}))}^{2}}$$

where *N* represents the number of atoms, *t_ref_* is the reference time, *r*′ denotes the coordinates of atoms at time *t_x_* after least-squares superposition on the reference frame, and *t_x_* is the simulation time of the corresponding analyzed frame.

Protein Cα RMSD values within 1–3 Å were considered indicative of stable protein structures, while ligand RMSD was used to assess binding retention throughout the simulation.

### 2.7. Experimental Validation

#### 2.7.1. MTT Viability Assay

Murine macrophage RAW 264.7 cells and Human Dermal Fibroblast (HDF) cells were obtained from the Skin Biotechnology Center at Kyung Hee University (Suwon, Republic of Korea). RAW264.7 cells (3 × 10^5^ cells/mL) and Human Dermal Fibroblast (HDF) cells (1 × 10^5^ cells/mL) were individually seeded into 96-well plates. The cells were stabilized by incubation for 24 h at 37 °C in a 5% CO_2_ incubator (MCO-18AIC, SANYO, Osaka, Japan). The culture medium was then removed, and the cells were re-incubated for another 24 h in a CO_2_ incubator after adding 180 μL of FBS-free DMEM containing 1% P/S and 20 μL of the sample diluted to the appropriate concentration. Following the incubation period, the attached cells were treated for 30 min with a solution prepared by mixing 20 μL of 5 mg/mL MTT (3-(4,5-dimethylthiazol-2-yl)-2,5-diphenyltetrazolium bromide) solution with 980 μL of serum-free medium. The MTT solution was then removed, and 100 μL of DMSO was added. The plate was shaken for 1 min on a plate shaker (Micromixer Mx4, FinePCR, Gunpo, Republic of Korea), and the absorbance was measured at 550 nm (Epoch, BioTek, Winooski, VT, USA). The results were calculated as cell viability (%) relative to the untreated control group.

#### 2.7.2. Radical Scavenging Assay

The antioxidant capacity of *T. flammea* extract was assessed using ABTS and DPPH radical scavenging assays following the methods of Re et al. and Blois, respectively, with minor modifications [38,39]. For the ABTS assay, the ABTS•^+^ radical cation was generated by mixing 14 mM ABTS solution (prepared in 1× PBS) with 4.9 mM potassium persulfate at a 1:1 (*v*/*v*) ratio and allowing the reaction to proceed in the dark at room temperature for 12 h. The resulting ABTS•^+^ solution was diluted with PBS to achieve an absorbance of 0.70 ± 0.10 at 734 nm. Subsequently, 10 μL of the extract, diluted to the designated concentrations in PBS, was mixed with 190 μL of the ABTS•^+^ working solution in a 96-well plate. After 6 min incubation at room temperature, the absorbance was measured at 734 nm using a SpectraMax iD3 microplate reader (Molecular Devices, San Jose, CA, USA). Ascorbic acid (AA) served as a positive control, and PBS alone was used as the blank. The radical scavenging activity (%) was calculated relative to the control group. For the DPPH assay, a 0.3 mM DPPH solution was prepared in 80% methanol and equilibrated for 30 min in the dark prior to use. Each well received 10 μL of appropriately diluted sample and 190 μL of DPPH working solution. The mixture was incubated for 30 min in the dark at room temperature, and the absorbance was recorded at 517 nm using the same microplate reader. Ascorbic acid and 80% methanol were used as the positive and negative controls, respectively. The DPPH radical scavenging activity (%) was determined by comparing the absorbance of treated wells against that of the control.

#### 2.7.3. Anti-Inflammatory Activity Assay

The anti-inflammatory activity of *T. flammea* extract was evaluated in murine macrophage RAW 264.7 cells. Cells were seeded at a density of 3 × 10^5^ cells/mL in 12-well plates and incubated at 37 °C in a humidified atmosphere containing 5% CO_2_ for 24 h. After stabilization, the culture medium was replaced with FBS-free DMEM containing 1% penicillin–streptomycin (P/S) and the extract diluted to the indicated concentrations (0.1, 1, 10, and 100 µg/mL). Dexamethasone (20 µg/mL) was used as a positive control. Cells were pre-treated with the extract or positive control for 30 min, after which inflammation was induced by the addition of 10 μg/mL lipopolysaccharide (LPS). Following 24 h incubation, either cultured cell or culture supernatant were collected for subsequent analyses.

Levels of pro-inflammatory cytokines (IL-6 and TNF-α) were quantified using enzyme-linked immunosorbent assays (ELISA) according to the manufacturer’s instructions, and absorbance was measured at 450 nm using a microplate reader (Epoch, BioTek, Winooski, VT, USA). The inhibitory effect of the extract was expressed relative to the LPS-treated control group. Nitric oxide (NO) production in the same supernatants was determined using the Griess reagent, and absorbance was read at 540 nm to calculate the percentage inhibition of NO formation. The expression level of cyclooxygenase-2 (COX-2) was analyzed by quantitative real-time PCR (qRT-PCR). Total RNA was extracted using Easy-Blue™ reagent, and cDNA was synthesized from 1 μg of RNA with the iScript™ cDNA Synthesis Kit. qRT-PCR was performed using a Real-time PCR system (AriaMx, Agilent, Santa Clara, CA, USA), using the intercalating dye-based reagent TB Green Premix Ex Taq (Takara Bio, Kusatsu, Japan) with gene-specific primers for COX-2 and the internal control GAPDH. Relative expression levels were calculated by the 2^−^ΔΔCt method, and the results were compared between sample-treated and untreated control cells to assess the transcriptional suppression of COX-2.

#### 2.7.4. Anti-Photoaging Effect Assessment (MMP-1 Assay)

HDF cells (1 × 10^5^ cells/mL) were seeded into a 12-well plate and stabilized by incubation at 37 °C in a 5% CO_2_ incubator for 24 h. Subsequently, the culture medium was removed, and a wrinkle-inducing environment was established by adding 400 μL of HBSS and irradiating with UVB (20 mJ/cm^2^) in HBSS. The HBSS was then removed, and the cells were re-incubated for 24 h in the CO_2_ incubator after adding 900 μL of FBS-free DMEM medium containing 1% P/S and 100 μL of the sample (final concentrations: 0.1, 1, 10, and 100 µg/mL). Retinol (20 µM) was used as a positive control. Following the incubation period, the culture supernatant was collected, and the production level of MMP-1 was quantified by performing ELISA. The absorbance was measured at 450 nm using a microplate reader (Epoch, BioTek) and compared with the untreated control group.

## 3. Results

### 3.1. Metabolite Profiling of T. flammea Extract Using UHPLC-MS/MS

**Figure 3:**
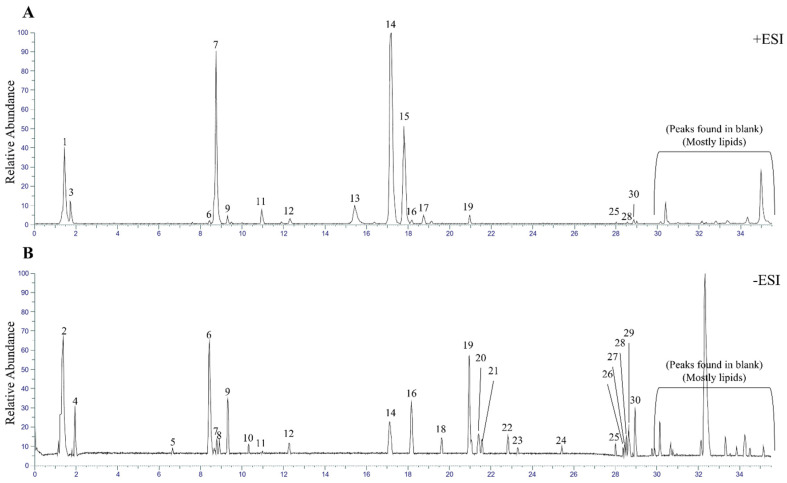
Base peak chromatograms of *T. flammea* extract using UHPLC-Orbitrap-MS/MS. (**A**) Positive ESI mode. (**B**) Negative ESI mode.

Comprehensive metabolomic profiling of the *T. flammea* ethanolic extract was performed using an UHPLC–Orbitrap ID-X Tribrid mass spectrometer to elucidate its major secondary metabolites. The sample (1 mg mL^−1^) was analyzed in both positive (+ESI) and negative (−ESI) ionization modes to ensure broad coverage of diverse chemical classes. Chromatographic separation was achieved within a 35 min gradient, and representative base-peak chromatograms (BPCs) are shown in Figure 3. The BPCs in both ion modes (Figure 3A,B) exhibited numerous well-resolved peaks, confirming the chemical complexity of the *T. flammea* extract and indicating the presence of structurally heterogeneous metabolites.

By comparison with procedural blanks, thirty significant peaks were selected for further interpretation. Data-dependent acquisition (DDA) was employed to obtain MS/MS spectra for each precursor ion, enabling fragmentation-based structural elucidation. High-resolution full-scan data (resolution = 120,000) combined with diagnostic product-ion analysis allowed tentative identification of 21 metabolites. Compound annotation was carried out by matching accurate masses, isotopic patterns, and fragmentation profiles against public and in-house databases. A complete list of the detected metabolites, including retention time, precursor *m*/*z*, molecular formula, ion mode, and putative compound name, is provided in Supplementary Table S1.

### 3.2. Identification and Classification of Putative Bioactive Metabolites

Untargeted UHPLC–MS/MS analysis of the *T. flammea* extract yielded a chemically heterogeneous metabolite profile encompassing both primary and secondary metabolites. To emphasize compounds of pharmacological interest, low-molecular-weight primary metabolites such as amino acids, simple organic acids, and fatty acid derivatives were excluded from subsequent evaluation. After this refinement, seventeen metabolites were confidently attributed to *T. flammea* with Level 2a identification confidence (probable structures supported by accurate mass, MS/MS fragmentation, and spectral library or literature comparison) (Table 1).

The annotated metabolites were grouped into three dominant structural classes: (i) flavonoids, (ii) pyrrolizidine alkaloids, and (iii) quinic acid–derived phenylpropanoids. Within these categories, the pyrrolizidine alkaloids (Lindelofidine, Angeloylplatinecine, Rivularine, and Thesinine) and phenolic compounds (Chlorogenic acid and Kaempferol rutinoside) represented the predominant constituents, as indicated by their strong chromatographic intensities and diagnostic production fragments. Additional minor flavonoids and alkaloids were detected at lower relative abundance, suggesting potential synergistic or modulatory contributions to the overall bioactivity of the extract. Because of isomeric overlap and limited chromatographic resolution under the present analytical conditions, unambiguous differentiation of possible thesinine isomers and hydroxylated derivatives could not be achieved.

### 3.3. Identification of Mechanistic Targets Involved in Skin Aging

**Figure 4:**
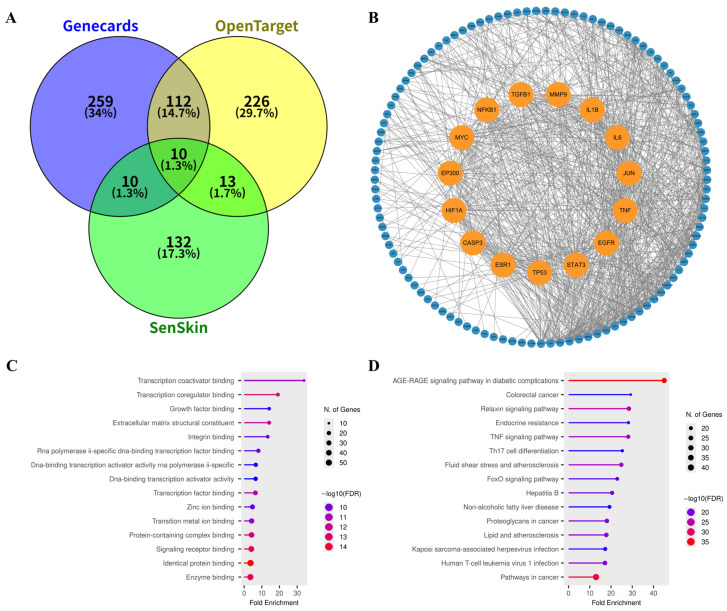
Network profile of skin aging-related genes. (**A**) Venn diagram showing the intersection between disease-associated targets obtained from the GeneCards (blue), Open Targets (yellow), and SenSkin (green) databases. (**B**) Protein–protein interaction (PPI) network of 145 intersecting genes, highlighting the top 15 hub genes in orange. (**C**) Gene Ontology (GO) molecular function (MF) enrichment analysis of the 145 intersecting genes. (**D**) KEGG pathway enrichment analysis of the 145 intersecting genes.

To identify molecular targets mechanistically associated with skin aging, comprehensive gene mining was performed using the GeneCards and Open Targets databases with the keyword “skin aging.” In addition, the manually curated SenSkin repository was consulted to include genes experimentally linked to cutaneous senescence. After removal of duplicates, a total of 762 non-redundant targets were collected (Supplementary Table S2). Among these, 145 genes were concurrently listed in at least two independent databases (Figure 4A), representing high-confidence candidates consistently associated with skin-aging phenotypes.

A protein–protein interaction (PPI) network was subsequently constructed from the 145 overlapping genes using the STRING v11.5 platform (high confidence ≥ 0.7) (Figure 4B, Supplementary Table S3). Topological analysis revealed that the resulting skin-aging interactome is organized around several densely connected hubs. Based on degree centrality, the top fifteen nodes included pro-inflammatory cytokines (TNF, IL6, IL1B), transcriptional regulators (JUN, NFKB1, MYC, HIF1A, ESR1), growth factors and their receptors (EGFR, TGFB1), and apoptosis-related mediators (TP53, CASP3). These hub proteins represent key convergence points that coordinate inflammation, oxidative stress response, cellular senescence, and extracellular-matrix remodeling during cutaneous aging.

Functional enrichment analysis was further performed on the 145 overlapping targets using the ShinyGO v0.85 platform. In total, 1822 significantly enriched terms were obtained—comprising 1000 biological processes (BP), 186 cellular components (CC), 434 molecular functions (MF), and 202 KEGG pathways (Supplementary Table S4). The top fifteen enriched categories were visualized (Figure 4C,D and Figure S1). GO analysis highlighted key processes associated with transcription factor binding, ECM structural organization, metal ion binding, and receptor-mediated signaling (Figure 4C). Correspondingly, KEGG enrichment indicated strong representation of the AGE–RAGE and TNF signaling pathways, both of which are critically involved in oxidative stress responses, pro-inflammatory activation, and collagen turnover in senescent skin (Figure 4D).

### 3.4. Identification of Potential Functional Targets of T. flammea Metabolites

**Figure 5:**
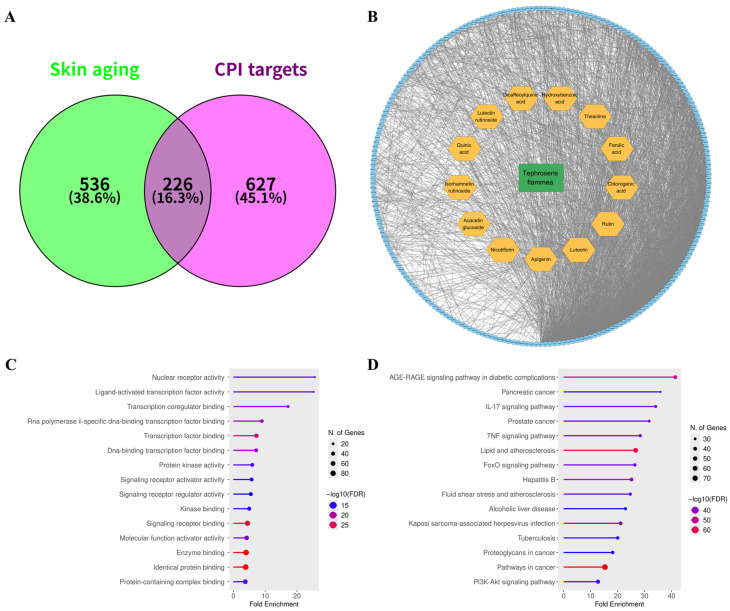
Network pharmacology to analyze *T.flammea* extract for skin aging. (**A**) Venn diagram showing the intersection between compound-associated targets (green) and non-redundant disease-associated targets collected from databases (magenta). (**B**) Compound–protein interaction (CPI) and protein–protein interaction (PPI) network of 13 *T.flammea* secondary metabolites and 226 intersecting genes. (**C**) Gene Ontology (GO) molecular function (MF) enrichment analysis of the 226 intersecting genes. (**D**) KEGG pathway enrichment analysis of the 226 intersecting genes.

By integrating experimentally reported and computationally inferred compound–protein interaction data, a total of 1596 distinct interaction pairs were identified, encompassing 853 non-redundant protein targets associated with 13 secondary metabolites detected in the *T. flammea* extract. These associations were compiled from multiple sources, including PubChem, CTD, DGIdb, BindingDB, SwissTargetPrediction, and STITCH, to ensure comprehensive coverage of validated and predicted molecular interactions (Supplementary Tables S5–S8). Cross-referencing these targets with the predefined skin-aging gene set yielded 226 overlapping genes (Figure 5A, Supplementary Table S9), representing putative functional mediators through which *T. flammea* metabolites may exert anti-aging effects.

A compound–protein interaction (CPI) network was subsequently constructed using Cytoscape v3.10.4, with node degree and betweenness centrality employed to evaluate topological importance (Figure 5B). The resulting network exhibited a characteristic multi-compound–multi-target configuration, consistent with the synergistic and complementary pharmacology typically observed in natural product systems. Among the detected metabolites, flavonoids such as apigenin, luteolin, and rutin displayed the highest target connectivity, largely reflecting their well-documented biological promiscuity and extensive prior characterization. Chlorogenic acid likewise showed broad interaction coverage, consistent with its established pharmacological versatility. In contrast, Thesinine—a comparatively understudied pyrrolizidine alkaloid—demonstrated potential interactions with a diverse set of targets, albeit with lower evidence scores. This apparent limitation may reflect the limited experimental data available for pyrrolizidine derivatives rather than an absence of bioactivity. Accordingly, Thesinine and related alkaloids were considered as alternative yet promising contributors to the overall functional profile of *T. flammea*, warranting further experimental evaluation in subsequent mechanistic studies.

### 3.5. Network Pharmacology and Pathway Enrichment Analysis

Analysis of the PPI network constructed from the 226 intersecting targets using the STRING v12.0 database (confidence score ≥ 0.7, Supplementary Table S10) revealed several nodes central to inflammation, oxidative stress regulation, and extracellular matrix (ECM) maintenance. The top ten hub proteins, ranked by degree centrality, were AKT1, TNF, IL6, TP53, IL1B, JUN, STAT3, HIF1A, EGFR, and CASP3—key regulators governing cellular survival, signaling, and structural homeostasis through the PI3K–AKT, STAT3, and ECM-remodeling pathways. Meanwhile, targets with the highest quantity of compound–protein interaction (CPI) link evidence—TNF, CASP3, IL1B, IL6, RELA, PTGS2, MAPK1, NFE2L2, MAPK3, and CAT—reflected overlapping biological functions, primarily related to inflammatory signaling, apoptotic control, and oxidative defense.

To clarify the biological functions and signaling relevance of these targets, Gene Ontology (GO) and Kyoto Encyclopedia of Genes and Genomes (KEGG) enrichment analyses were conducted using the ShinyGO v0.85 platform with a false discovery rate (FDR) threshold of <0.05 (Figure 5C,D and Figure S2). In total, over 1000 biological processes (BP), 308 cellular components (CC), and 674 molecular functions (MF) were significantly enriched (Supplementary Table S11). GO–BP terms were dominated by processes linked to oxidative and chemical stress responses, lipid-mediated and prostaglandin signaling, apoptosis regulation, and intercellular communication—hallmarks of skin-aging physiology. Enriched CC categories included extracellular matrix organization, cytoplasmic vesicles, and nuclear compartments, suggesting that *T. flammea* metabolites influence both intracellular signaling and extracellular structural remodeling. MF enrichment emphasized binding activities of signaling receptors, kinases, and transcription factors, reflecting multitarget modulation of signal transduction and redox homeostasis.

KEGG pathway analysis identified 229 significantly enriched signaling pathways (Supplementary Table S11). Among them, canonical aging- and inflammation-related pathways such as AGE–RAGE, IL-17, EGFR, and TNF signaling were prominently represented. Pathways associated with cancer types—including bladder, pancreatic, prostate, and colorectal cancers—also appeared frequently, likely reflecting shared molecular underpinnings between chronic inflammation, ECM degradation, and carcinogenesis. Overall, this multi-layered network and pathway enrichment analysis delineates the polypharmacological potential of *T. flammea*, highlighting its capacity to concurrently regulate oxidative balance, inflammatory activity, and ECM integrity through an interconnected set of signaling pathways relevant to skin aging.

### 3.6. Molecular Docking and Dynamics Validation

#### 3.6.1. Molecular Docking

**Figure 6:**
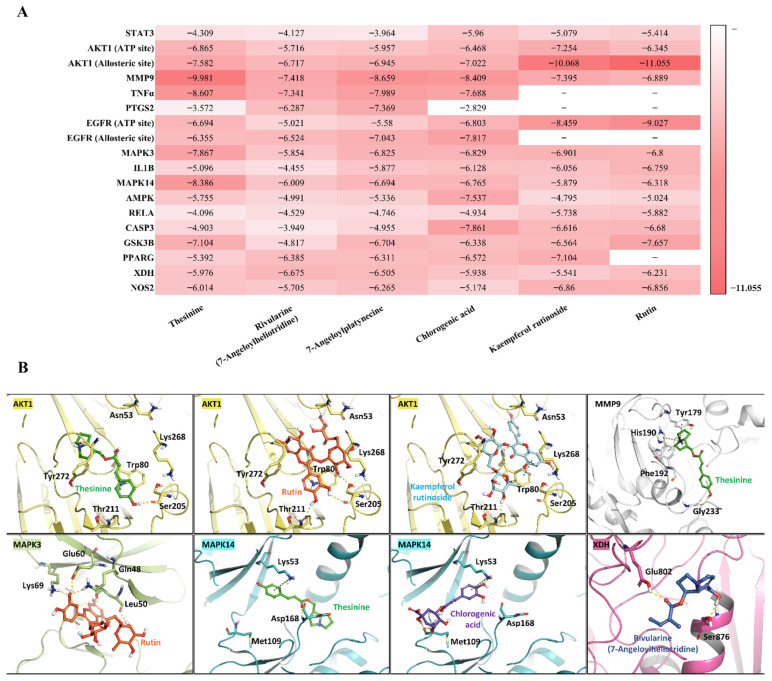
Molecular docking analysis of selected phytochemicals with key target proteins. (**A**) Heatmap of Glide docking gscores (kcal mol^−1^) for seven representative natural compounds docked against 18 protein targets. (**B**) Representative binding poses of Thesinine, Rutin, Kaempferol rutinoside, Chlorogenic acid, and Rivularine within five major targets exhibiting favorable interaction profiles. AKT1, MMP9, MAPK3, MAPK14, and XDH are shown as yellow, white, light green, cyan, and pink ribbon structures, respectively. The ligands are displayed as sticks in green (Thesinine), orange (Rutin), cyan (Kaempferol rutinoside), purple (Chlorogenic acid), and blue (Rivularine). Key residues involved in hydrogen bonding and hydrophobic interactions are labeled.

To further substantiate the findings from the network pharmacology analysis, molecular docking and molecular dynamics (MD) simulations were conducted to evaluate the binding behavior and dynamic stability of *T. flammea*-derived natural compounds with the predicted protein targets. Molecular docking was performed between six *T. flammea*-derived natural compounds and sixteen target proteins identified from the integrated protein–compound network (Figure 6A). Because AKT1 and EGFR possess distinct regulatory pockets, both the ATP-binding and allosteric sites were evaluated to capture potential differences in ligand accommodation. From these docking results, we selected protein–ligand pairs with the most favorable energy scores and binding poses consistent with reference co-crystal ligands. These complexes were then subjected to MD simulations to evaluate the stability of the predicted binding modes. For targets such as RELA, whose available structure comprises mainly the DNA-binding domain and lacks a co-crystallized ligand, the reliability of binding site prediction is inherently limited. Among the evaluated complexes, five target proteins—AKT1, MMP9, MAPK3, MAPK14, and XDH—showed relatively strong predicted binding affinities with five representative compounds (Thesinine, Rutin, Kaempferol rutinoside, Chlorogenic acid, and Rivularine) (Figure 6B). The calculated Glide gscores (−7.0 to −11.0 kcal mol^−1^) indicate energetically favorable binding, consistent with the presence of stable hydrogen bonds and hydrophobic interactions within their respective binding pockets.

#### 3.6.2. MD Simulation

**Figure 7:**
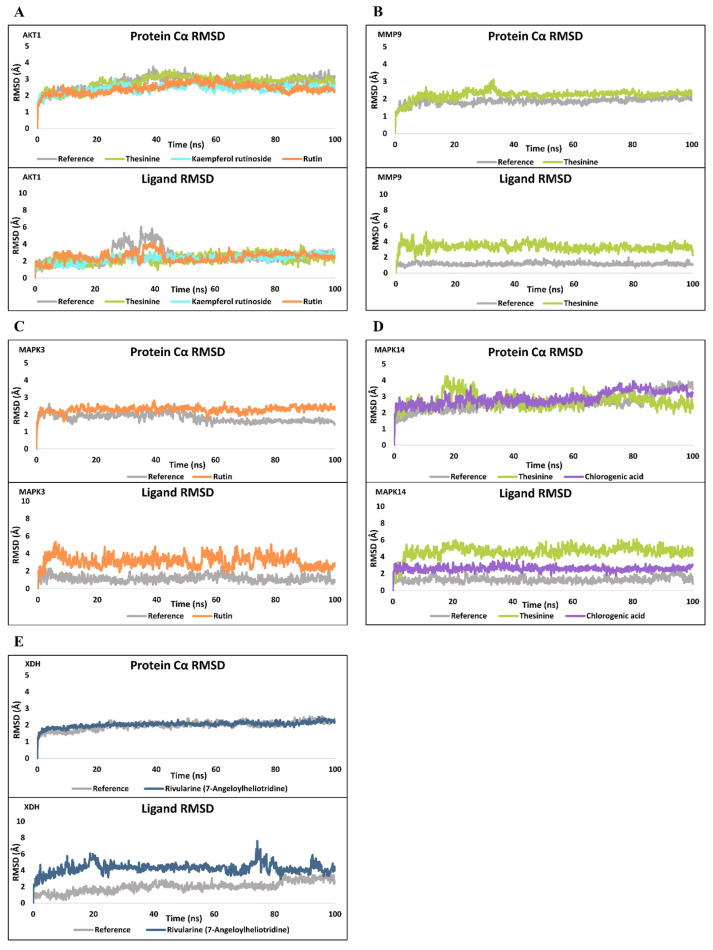
Molecular dynamics simulation results of three compounds bound to target proteins (replicate 1). Time-dependent root-mean-square deviation (RMSD) plots of protein Cα atoms and bound ligands during 100 ns MD simulations are shown for each target–ligand complex. The “reference” trace corresponds to the co-crystallized ligand from each protein structure. Thesinine, Rutin, Kaempferol rutinoside, Chlorogenic acid, and Rivularine are depicted as green, orange, cyan, purple, and deep blue trajectories, respectively. (**A**) AKT1. (**B**) MMP9. (**C**) MAPK3. (**D**) MAPK14. (**E**) XDH.

To evaluate the temporal stability of these predicted complexes, eight representative compound–protein pairs were subjected to 100 ns MD simulations, performed in triplicate for statistical reliability. Across all replicates, the protein Cα RMSD values remained within approximately 3 Å, indicating that the overall protein backbones were conformationally stable throughout the trajectories (Figure 7, Figures S3 and S4). In contrast, the ligand RMSD results highlighted compound-specific variations in binding persistence. The ligands bound to AKT1, MMP9, and MAPK3 maintained RMSD values below 5 Å in all three replicates (Figure 7A–C, Figures S3A–C and S4A–C), suggesting sustained and tight association within the active site pockets. These stable interactions were further corroborated by consistent protein–ligand contact maps, showing recurring hydrogen bonding and hydrophobic residues maintained for more than 30% of the simulation time (Supplementary Figure S5A–E). For MAPK14, the results revealed differential stability between the two ligands. The ligand RMSD of Chlorogenic acid remained consistently below 5 Å across all replicates, indicating a tightly maintained binding pose throughout the 100 ns simulations. In contrast, Thesinine displayed larger RMSD fluctuations exceeding 6–8 Å, suggesting increased mobility and partial dissociation from the active site (Figure 7D, Figures S3D and S4D). These trends were consistent with the protein–ligand contact profiles, where Chlorogenic acid formed persistent hydrogen bonds and hydrophobic contacts, whereas Thesinine exhibited weaker and less frequent interactions (Supplementary Figure S5F,G).

Unlike the other systems, the XDH–Rivularine complex displayed pronounced instability. In replicate 1, the ligand RMSD fluctuated moderately, whereas in replicate 2, values intermittently approached 20 Å, and in replicate 3, RMSD increased sharply to >100 Å after 70 ns (Figure 7E, Figures S3E and S4E). Corresponding contact-map analyses confirmed a rapid loss of hydrogen bonding and hydrophobic contacts (Supplementary Figure S5H), implying that the initially favorable docking conformation was not maintained dynamically. These results indicate that Rivularine fails to sustain stable binding within the XDH active site.

Collectively, the combined docking and MD simulation analyses demonstrate that several *T. flammea*-derived phytochemicals form stable and persistent interactions with AKT1, MMP9, MAPK3, and MAPK14, whereas their interaction with XDH is likely unstable under physiological conditions. These findings provide a mechanistic validation of the network-based predictions and highlight the potential of *T. flammea* metabolites as multi-target modulators of related pathways.

### 3.7. Experimental Validation

#### 3.7.1. Cell Viability Assessment

**Figure 8:**
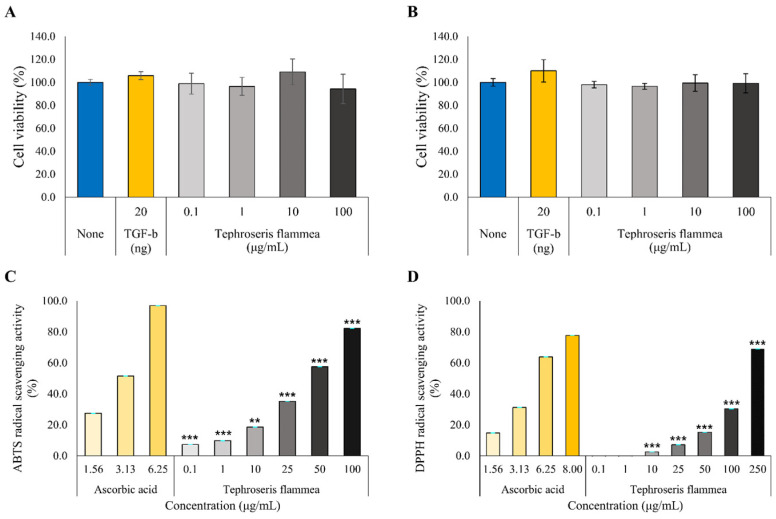
Effects of *T. flammea* extract on cell viability and free radical scavenging. Data are expressed as the mean ± S.D of three replicates. *** *p* < 0.001 and ** *p* < 0.01 as compared to none. (**A**) Effects of *T. flammea* extract on RAW264.7 cell viability. (**B**) Effects of *T. flammea* extract on human dermal fibroblasts (HDFs) cell viability. (**C**) ABTS radical scavenging activity of *T. flammea* extract. (**D**) DPPH radical scavenging activity of *T. flammea* extract.

Prior to evaluating the anti-inflammatory (in RAW264.7 cells) and anti-wrinkle (in HDFs) effects of the *T. flammea* extract, an MTT assay was performed to determine its effect on cell viability. RAW264.7 cells were treated with the *T. flammea* extract at concentrations of 0.1, 1, 10, and 100 µg/mL for 24 h. As shown in Figure 8A, cell viability was maintained or even slightly increased at low-to-mid concentrations, with viability percentages of 98.9% (0.1 µg/mL, *p* < 0.05), 96.5% (1 µg/mL), 109.1% (10 µg/mL, *p* < 0.01) and 94.3% (100 µg/mL, *p* < 0.001) compared to the 100% untreated control. When HDFs were treated with *T. flammea* extract at 0.1, 1, 10 µg/mL and 100 µg/mL cell viability was maintained at 98.0%, 96.9% (*p* < 0.05), 99.4% and 99.1% (*p* < 0.05), respectively (Figure 8B). Based on these results, the *T. flammea* extract was confirmed to be non-toxic to RAW264.7 macrophages and HDFs up to 100 µg/mL.

#### 3.7.2. Antioxidant Activity Assessment

To evaluate the antioxidant efficacy of the *T. flammea* extract, the representative radical scavenging assays, ABTS and DPPH, were performed. Ascorbic acid was used as a positive control.

The *T. flammea* extract effectively scavenged ABTS radicals in a concentration-dependent manner (Figure 8C). The scavenging activity significantly increased as the concentration rose: 7.3% (0.1 µg/mL), 9.8% (1 µg/mL), 18.6% (10 µg/mL), 35.2% (25 µg/mL), 57.6% (50 µg/mL) and 82.3% (100 µg/mL) (*p* < 0.001 for all concentrations). The IC50 value for the *T. flammea* extract was calculated to be 53.49 µg/mL. Although this value is numerically higher than that of the positive control, Ascorbic acid (IC50: 3.03 µg/mL), it indicates that the *T. flammea* extract possesses potent ABTS radical scavenging activity, given that it is a complex crude mixture.

In the DPPH radical scavenging activity assay (Figure 8D), the *T. flammea* extract also demonstrated a concentration-dependent antioxidant effect, though it was less potent than in the ABTS assay. The extract began to show significant scavenging activity from 10 µg/mL (2.6%) upwards, reaching 69.0% at 250 µg/mL (*p* < 0.001 at concentrations from 10 µg/mL upwards). The IC50 value for the *T. flammea* extract was calculated to be 160.02 µg/mL. In comparison, the positive control, Ascorbic acid, showed stronger activity with an IC50 value of 4.51 µg/mL.

Collectively, these results confirm that the *T. flammea* extract possesses concentration-dependent in vitro antioxidant activity. The IC50 values of the TF extract (ABTS: 53.49 µg/mL; DPPH: 160.02 µg/mL) were significantly higher than those of the pure compound Ascorbic acid (ABTS: 3.03 µg/mL; DPPH: 4.51 µg/mL). However, this is an expected comparison between a crude extract which contains a large mass of non-antioxidant components and a pure single compound. The fact that the crude extract exhibited these IC50 values in its unrefined state suggests that the active constituents within *T. flammea* possess strong antioxidant potential.

#### 3.7.3. Anti-Inflammatory Activity Assessment

**Figure 9:**
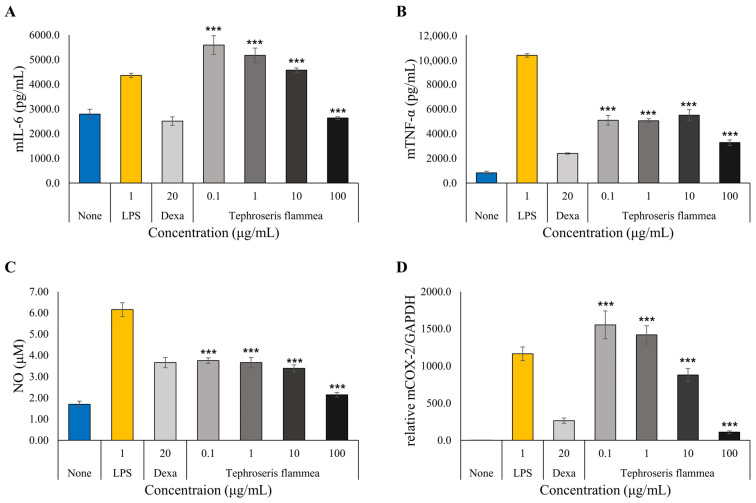
Anti-inflammatory effects of *T. flammea* on lipopolysaccharide (LPS)-stimulated RAW264.7 macrophages, demonstrating the inhibition of pro-inflammatory mediators. Data are expressed as the mean ±S.D of three replicates. *** *p* < 0.001 as compared to the LPS (1 μg/mL) group. (**A**) TNF-α. (**B**) IL-6. (**C**) NO. (**D**) PTGS2 (COX-2).

To evaluate the anti-inflammatory activity of *T. flammea* extract, RAW 264.7 macrophages were stimulated with 1 μg/mL lipopolysaccharide (LPS) to induce an inflammatory state. Dexamethasone (Dexa, 20 μg/mL) was used as a positive control due to its well-established potency in inhibiting pro-inflammatory cytokines and mediators in this cellular model [40,41], providing a robust benchmark for efficacy. The *T. flammea* extract effects on key pro-inflammatory cytokines and mediators were assessed (Figure 9). LPS treatment alone markedly increased the production of all inflammatory markers compared to the untreated None group, confirming the successful induction of inflammation.

Inhibition of TNF-α and Nitric oxide (NO) production *T. flammea* extract demonstrated a potent and consistent inhibitory effect on the production of the key upstream mediators TNF-α and NO (Figure 9A,C).

TNF-α (Figure 9A) was used as LPS treatment, significantly increasing TNF-α levels to 10,348.2 pg/mL from a baseline of 826.5 pg/mL. The *T. flammea* extract significantly inhibited this production in a clear dose-dependent manner at all tested concentrations: 5093.9 pg/mL (0.1 µg/mL), 5047.3 pg/mL (1 µg/mL), 5499.4 pg/mL (10 µg/mL), and 3279.6 pg/mL (100 µg/mL) (all *p* < 0.001 vs. LPS). The effect at 100 μg/mL was comparable to the Dexa-treated group (2394.9 pg/mL).

Nitric oxide (NO) (Figure 9C) was used as LPS treatment, increasing NO levels from 1.70 μM to 6.15 μM. The *T. flammea* extract also showed significant, dose-dependent inhibition at all concentrations: 3.75 μM (0.1 µg/mL), 3.66 μM (1 µg/mL), 3.39 μM (10 µg/mL), and 2.14 μM (100 µg/mL) (all *p* < 0.001 vs. LPS). Notably, at concentrations of 1 μg/mL and higher, *T. flammea* extract inhibitory effect was stronger than that of Dexa (3.66 μM).

The biphasic modulation of IL-6 and COX-2 occurred. In contrast to TNF-α and NO, *T. flammea* extract exhibited a complex, biphasic (hormetic) effect on IL-6 and COX-2 expression (Figure 9B,D).

IL-6 (Figure 9B) was used as LPS stimulation, raising IL-6 levels to 4361.3 pg/mL. At low-to-mid concentrations (0.1, 1, and 10 µg/mL), *T. flammea* extract significantly increased IL-6 production to 5594.1, 5176.4, and 4575.3 pg/mL, respectively. However, at the high concentration of 100 μg/mL, the extract significantly suppressed IL-6 production to 2636.1 pg/mL (*p* < 0.001 vs. LPS), a level comparable to the Dexa control (2508.2 pg/mL).

COX-2 mRNA expression (Figure 9D) displayed a similar biphasic pattern, which was increased to a relative level of 1163.1 by LPS. The *T. flammea* extract significantly increased expression at 0.1 μg/mL (1551.7) and 1 μg/mL (1417.6). Conversely, it significantly suppressed COX-2 expression at 10 μg/mL (880.3) and most potently at 100 μg/mL (109.6) (*p* < 0.001 vs. LPS), showing a stronger suppression than Dexa (265.0).

#### 3.7.4. Anti-Photoaging Effect Assessment: Inhibition of MMP-1 Production

**Figure 10:**
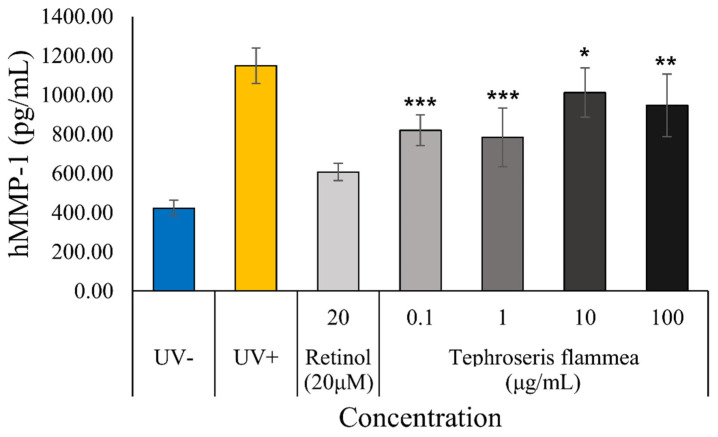
Inhibitory effect of *T. flammea* extract on UVB-induced MMP-1 production in human dermal fibroblasts (HDFs). Data are expressed as the mean ± S.D of three replicates. *** *p* < 0.001, ** *p* < 0.01 and * *p* < 0.05 compared to the UV+ group.

The anti-photoaging potential of *T. flammea* extract was evaluated in UVB-irradiated human dermal fibroblasts (HDFs). The effect was quantified by measuring the production of matrix metalloproteinase-1 (MMP-1), a key collagen-degrading enzyme, via ELISA (Figure 10).

UVB exposure (20 mJ/cm^2^) successfully induced photoaging, markedly increasing MMP-1 secretion from a baseline of 423.42 pg/mL (UV- group) to 1149.42 pg/mL (UV+ group). The positive control, Retinol (20 µM), significantly suppressed this increase to 606.96 pg/mL (*p* < 0.001 vs. UV+).

The *T. flammea* extract exhibited a significant and complex, non-linear dose–response against MMP-1 production. At low-to-mid concentrations, the extract showed potent inhibition, reducing MMP-1 levels to 820.55 pg/mL (0.1 µg/mL, *p* < 0.001) and achieving a maximal suppression at 1 μg/mL (783.96 pg/mL, *p* < 0.001).

However, as the concentration increased further to 10 μg/mL and 100 μg/mL, the inhibitory effect partially reversed, with MMP-1 levels rising again to 1012.08 pg/mL (*p* < 0.05) and 946.95 pg/mL (*p* < 0.01), respectively. Although all tested concentrations remained statistically significant in suppressing MMP-1 compared to the UV+ control, the strongest biological effect was clearly observed at the low-mid concentration range (0.1–1 µg/mL). These findings demonstrate that *T. flammea* extract possesses potent anti-photoaging capabilities by inhibiting UVB-induced MMP-1 production, particularly at lower concentrations.

## 4. Discussion

This study provides a comprehensive demonstration of how an informatics-integrated, multi-layered analytical framework can effectively elucidate the anti-aging potential of previously uncharacterized botanical species. By combining untargeted UHPLC–MS/MS metabolomics, network pharmacology, molecular docking and dynamics, and in vitro validation, we systematically identified both the chemical constituents and mechanistic targets of *Tephroseris flammea*. The integrated results revealed that 21 secondary metabolites—predominantly flavonoids, phenylpropanoids, and pyrrolizidine alkaloids—collectively modulate a network of 226 skin-aging–related targets. Among these, AKT1, RELA, and MAPK3 emerged as principal signaling hubs governing oxidative stress response, inflammatory activation, and extracellular matrix remodeling. The stable binding interactions predicted for these proteins were substantiated through 100 ns molecular-dynamics simulations, in which selected compounds such as chlorogenic acid, kaempferol-O-rutinoside, and thesinine maintained consistent hydrogen bonding and hydrophobic contacts within their active pockets. Experimental bioassays further corroborated the in silico predictions: the *T. flammea* extract exhibited strong radical-scavenging capacity in both ABTS and DPPH assays and significantly suppressed UVB-induced IL-6, TNF-α, COX-2, and MMP-1 expression in fibroblast and macrophage models without detectable cytotoxicity.

A notable non-linear response was observed for the downstream mediators IL-6, COX-2, and MMP-1. For the inflammatory mediators IL-6 and COX-2, this biphasic response can be attributed to a hormetic effect commonly seen in phytochemistry, where low-dose phytochemicals may act as mild cellular primers [42,43]. Specifically, this phenomenon (often termed xenohormesis) suggests that sub-toxic concentrations of secondary metabolites can trigger adaptive stress response pathways, resulting in mild immunostimulation, whereas higher concentrations exert the potent inhibitory effects required for therapeutic efficacy. However, the response of MMP-1, which was more strongly inhibited at a lower concentration (1 µg/mL) than at 100 µg/mL level, requires a different explanation. It is likely that the consistently suppressed upstream mediators—TNF-α and NO—inhibit MMP-1 expression, as expected [44,45], but an unexpected secondary mechanism triggered by the extract at a high concentration partially counteracts this effect. As our network pharmacology identified a large set of potential protein interactors (Supplementary Table S9), it is plausible that at high concentrations, extract components begin to activate unintended, low-affinity “off-targets” including MAPKs [46]. The activation of such a secondary pathway could provide a competing signal that partially reverses the suppression of MMP-1. Nevertheless, it is critical that the key upstream mediators, TNF-α and NO, were consistently and dose-dependently inhibited at all tested concentrations. Furthermore, the extract remained a statistically significant MMP-1 inhibitor even at its highest concentration (100 µg/mL) compared to the UVB-irradiated control. The coherence between computational and biological data highlights the robustness of this multi-tiered approach, confirming that *T. flammea* contains multifunctional metabolites capable of simultaneously targeting oxidative, inflammatory, and matrix-degrading pathways central to cutaneous aging.

The mechanistic landscape of *T. flammea* converges established antioxidant pathways with unique alkaloid-driven features. While flavonoids and phenolic acids—such as kaempferol-O-rutinoside and chlorogenic acid—stabilize the ECM via PI3K–AKT and MAPK signaling [47,48,49], *T. flammea* is distinguished by its substantial content of the pyrrolizidine alkaloid Thesinine [50]. Unlike classical radical-scavenging flavonoids, docking simulations suggest Thesinine acts as a dual modulator of redox-sensitive kinases, stabilizing the AKT1 ATP-binding cleft and anchoring to MAPK3 residues. This complementarity between flavonoid antioxidants and alkaloid kinase modulators distinguishes *T. flammea* from polyphenol-dominant botanicals like Paeonia lactiflora or Hibiscus mutabilis [51,52]. Chemically, this simultaneous accumulation of alkaloids and phenolics represents a distinctive biosynthetic adaptation within the Asteraceae family, broadening the extract’s pharmacophore coverage beyond simple antioxidant effects. Consequently, the extract coordinates the regulation of AKT–NF-κB and MAPK axes, providing a computationally validated and biologically safe model for targeting the oxidative and inflammatory drivers of skin aging.

The absence of cytotoxicity further underscores its biocompatibility and suitability for dermal applications. These findings demonstrate that the integrative pipeline used here not only accelerates the identification of bioactive plant species but also provides mechanistic clarity linking chemical identity to functional outcome. In this context, *T. flammea* represents both a pharmacologically relevant and computationally validated model for developing multifunctional natural agents targeting oxidative and inflammatory aspects of skin aging.

First and foremost is the toxicological concern regarding pyrrolizidine alkaloids (PAs), a major metabolite class identified in the *T. flammea* extract [53]. It is crucial, however, to differentiate between PA subclasses based on their chemical structure. The well-documented hepatotoxicity and genotoxicity of PAs are primarily associated with 1,2-dehydropyrrolizidine (unsaturated) esters—such as rivularine (7-angeloylheliotridine, also detected in our extract) or senecionine—which are metabolically activated by hepatic CYP450 enzymes into reactive pyrrolic esters [53,54,55,56]. In sharp contrast, Thesinine, one of the most abundant metabolites in our profile, is a saturated PA (an otonecine-type ester). Saturated PAs lack the 1,2-double bond necessary for this toxic metabolic activation pathway and are therefore generally regarded as non-toxic or possessing significantly lower toxicity [57,58]. Furthermore, *T. flammea* conjugates like thesinine-4′-O-glucoside are similarly considered safe [59]. Ethnobotanically, the young leaves of *T. flammea* have been traditionally consumed as a seasonal vegetable (“Namul”) in Korea, suggesting a history of human tolerance to its oral ingestion. Consistent with this, the *T. flammea* extract, whose profile is dominated by saturated PAs, did not induce significant cytotoxicity or apoptosis in our in vitro models. While this does not preclude systemic toxicity, it suggests the extract itself is not overtly cytotoxic to dermal cells. Nonetheless, the presence of any unsaturated PA (e.g., Rivularine) poses a significant regulatory and safety hurdle. Therefore, future development strategies must involve optimized extraction or chromatographic purification to selectively eliminate toxic unsaturated PAs while retaining bioactive, non-toxic constituents.

In the technical perspective, our quantitative UHPLC–MS/MS dataset and computational analyses already provide robust chemical and mechanistic coverage; thus, the current limitations do not arise from the in silico domain but rather from the experimental resolution at the compound level. The biological validation in this work was performed primarily using the crude extract, which accurately reflects the native biochemical synergy but limits mechanistic interpretation for individual constituents. Because our workflow follows a “from-selection-to-validation” sequence, detailed characterization of isolated metabolites—such as determining dose–response behavior, molecular specificity, and potential synergism—will be an essential next phase to obtain in-depth mechanistic insight. Another limitation lies in the absence of in vivo evaluation. While the in vitro assays confirmed antioxidant, anti-inflammatory, and anti-photoaging efficacy without cytotoxicity, further studies are needed to examine safety, potential toxicity, and physiological specificity in animal or reconstructed human skin models.

Moreover, practical aspects related to industrial feasibility remain unexplored. As *T. flammea* is a wild species with limited ecological distribution, studies on its sustainable sourcing, cultivation potential, and biotechnological production routes will be crucial to ensure consistent material supply. Future investigations combining targeted isolation, in vivo pharmacological testing, and resource-management strategies will thus consolidate both the scientific and practical foundations for translating *T. flammea* into a viable natural ingredient for skin-aging intervention.

## 5. Conclusions

This study provides the first comprehensive characterization of *Tephroseris flammea*, integrating informatics, metabolomics, molecular modeling, and biological validation within a unified discovery framework. Through untargeted UHPLC–MS/MS profiling, 21 major metabolites—mainly flavonoids, phenylpropanoids, and pyrrolizidine alkaloids—were identified and linked to 226 skin-aging–related targets, with AKT1, RELA, and MAPK3 emerging as pivotal regulatory hubs. Docking and molecular-dynamics analyses confirmed stable compound–target interactions, while in vitro assays demonstrated significant antioxidant, anti-inflammatory, and anti-photoaging effects without cytotoxicity. The strong concordance between these computational predictions and experimental results primarily validates the utility of our integrative pipeline. This work thus serves as a methodological validation, demonstrating that our “from-selection-to-validation” workflow can successfully elucidate complex mechanisms even from uncharacterized plant taxa.

The identification of both saturated (e.g., Thesinine) and unsaturated (e.g., Rivularine) PAs highlights a critical toxicological challenge that must be addressed before this plant can be considered for commercial application. However, our findings also suggest a clear and rational path forward. Future research should focus on developing optimized extraction and purification methodologies—guided by our metabolomic data—to either (1) selectively remove the hazardous unsaturated PAs while retaining active constituents, or (2) isolate the abundant, likely non-toxic saturated PAs (like Thesinine) for individual assessment. This targeted approach, initiated by our in silico pipeline, would consolidate the potential of *T. flammea* as a valuable source for novel, safe, and mechanistically defined functional ingredient.

## Figures and Tables

**Table 1:** 17 major secondary metabolites identified in *T.flammea* extract. Compounds detected at relatively high abundance are indicated in bold.

**Molecule Name**	**RT**	**Monoisotopic Mass**	**Molecular** **Formula**	**Intensity** **(POS)**	**Intensity** **(NEG)**
Quinic acid	1.39	192.0634	C_7_H_12_O_6_	1.78 × 10^4^	4.61 × 10^6^
Lindelofidine (or its chiral isomer)	1.75	141.1154	C_8_H_15_NO	8.96 × 10^6^	ND
Hydroxybenzoic acid	6.66	138.0317	C_7_H_6_O_3_	1.23 × 10^3^	6.01 × 10^5^
Chlorogenic acid	8.43	354.0951	C_16_H_18_O_9_	1.55 × 10^6^	4.31 × 10^6^
Angeloylplatinecine	8.71	239.1521	C_13_H_21_NO_3_	6.76 × 10^7^	ND
Rivularine	8.78	237.1365	C_13_H_19_NO_3_	1.02 × 10^7^	8.51 × 10^5^
Thesin (thesinine dimer)	15.43	574.3043	C_34_H_42_N_2_O_6_	7.41 × 10^6^	3.61 × 10^3^
Thesinine	17.17	287.1521	C_17_H_21_NO_3_	7.26 × 10^7^	1.53 × 10^6^
Rutin	18.18	610.1534	C_27_H_30_O_16_	1.86 × 10^6^	2.20 × 10^6^
Luteolin rutinoside	19.64	594.1585	C_27_H_30_O_15_	5.87 × 10^5^	9.63 × 10^5^
Kaempferol rutinoside (Nicotiflorin)	20.96	594.1585	C_27_H_30_O_15_	3.64 × 10^6^	3.81 × 10^6^
Ferulic acid	21.41	194.0579	C_10_H_10_O_4_	ND	1.09 × 10^6^
Isorhamnetin rutinoside (Narcissin)	21.57	624.1690	C_28_H_32_O_16_	5.95 × 10^5^	9.02 × 10^5^
Dicaffeoylquinic acid	23.3	516.1267	C_25_H_24_O_12_	1.93 × 10^5^	6.18 × 10^5^
Acacerin glucoside (Tilianin)	28.02	446.1212	C_22_H_22_O_10_	9.81 × 10^5^	7.63 × 10^5^
Apigenin	28.38	270.0522	C_15_H_10_O_5_	2.41 × 10^5^	6.34 × 10^5^
Luteolin	28.47	286.0477	C_15_H_10_O_6_	1.12 × 10^5^	7.78 × 10^5^

ND: Not detected.

## Data Availability

Publicly available datasets were analyzed in this study. Disease–gene/protein relationship data were obtained from GeneCards (https://www.genecards.org/) and the Open Targets Platform (https://platform.opentargets.org/). Protein–protein interaction (PPI) data were retrieved from the STRING database (https://string-db.org/). Experimentally validated protein–chemical interaction (PCI) data were sourced from PubChem (https://pubchem.ncbi.nlm.nih.gov/), while bibliometric statistics for specific compounds were derived from PubMed (https://pubmed.ncbi.nlm.nih.gov/) and Springer Nature (accessed via PubChem). Prediction of PCIs was performed using SwissTargetPrediction (https://www.swisstargetprediction.ch/) and STITCH (http://stitch.embl.de/). Gene enrichment analysis was conducted using ShinyGO 0.85 (https://bioinformatics.sdstate.edu/go/). All data subsets retrieved and analyzed during the current study are available in the Supplementary Materials (Tables S2–S11), accessed on 7 January 2025.

## Supplementary Materials

The following supporting information can be downloaded at: https://www.mdpi.com/article/10.3390/biom15121740/s1. Supplementary Figure S1. Additional pathway enrichment analysis results involving 145 intersecting genes. (A) GO biological processes (BP)-based analysis. (B) GO cellular components (CC) -based analysis; Supplementary Figure S2. Additional pathway enrichment analysis results involving 226 *T. flammea* targets. (A) GO biological processes (BP)-based analysis. (B) GO cellular components (CC)-based analysis; Supplementary Figure S3. Molecular dynamics simulation results of three compounds bound to target proteins (replicate 2); Supplementary Figure S4. Molecular dynamics simulation results of three compounds bound to target proteins (replicate 3); Supplementary Figure S5. Protein–ligand contact profiles for three compounds bound to target proteins.; Supplementary Table S1. Full list of identified metabolites from *T. flammea* extract; Supplementary Table S2. Full list of skin aging-related genes identified from Genecards, Open Targets & SenSkinTM databases; Supplementary Table S3. Detailed information of PPI network consists of 145 intersecting genes; Supplementary Table S4. Full pathway enrichment analysis results involving 145 intersecting genes; Supplementary Table S5. Full list of *T. flammea* metabolite-protein association annotations collected from PubChem pre-compiled dataset; Supplementary Table S6. Full list of *T. flammea* metabolite-protein association annotations collected from PubChem bioassay dataset; Supplementary Table S7. Full list of *T. flammea* metabolite-protein association prediction results produced with SwissTargetPrediction; Supplementary Table S8. Full list of *T. flammea* metabolite-protein association prediction results produced with STITCH; Supplementary Table S9. Non-redundant list of *T. flammea* metabolite-protein association pairs identified from available databases; Supplementary Table S10. Detailed information of PPI network consists of 226 *T. flammea*-associated skin aging targets; Supplementary Table S11. Full pathway enrichment analysis results involving 226 *T. flammea* targets; Supplementary Table S12. Summary of protein target structures and corresponding PDB IDs used for molecular docking analysis.

## Abbreviations

The following abbreviations are used in this manuscript:

CPI	Compound–protein interaction
ECM	Extracellular matrix
GO	Gene ontology
HDF	Human dermal fibroblast
MD	Molecular dynamics
NP	Natural product
PPI	Protein–protein interaction
RMSD	Root mean square distance
ROS	Reactive oxygen species
UHPLC	Ultra high performance liquid chromatography
UV	Ultraviolet
