# Supplementary material for: An Integrated Systems Pharmacology Approach Combining Bioinformatics, Untargeted Metabolomics and Molecular Dynamics to Unveil the Anti-Aging Mechanisms of Tephroseris flammea
Source: Biomolecules. 2025 Dec 15;15(12):1740. doi: 10.3390/biom15121740 (PMC12730562; doi:10.3390/biom15121740)
Supplement: Supplementary file 1 [file biomolecules-15-01740-s001.zip › biomolecules-4015384-supplementary/Biomolecules_CHO_article_supplementary_material.pdf]

Supplementary material

An integrated systems pharmacology approach combining bioinformatics, untargeted metabolomics and molecular dynamics to unveil the anti-aging mechanisms of *Tephroseris flammea*

Min Hyung Cho[1*], Haiyan Jin[1], JangHo Ha[1,2], SungJune Chu[1], SoHee An[1]

1 Bioinformatics and Molecular Design Research Center (BMDRC). Songdogwahak-ro 85, Yeonsu-gu, In-cheon, 21983, Republic of Korea

2 Department of Integrative Biotechnology, Yonsei University, Songdogwahak-ro 85, Yeonsu-gu, Incheon, 21983, Republic of Korea

* Correspondence: Min Hyung Cho (mhcho@bmdrc.org)

Contents

Supplementary figures

Supplementary figure S1. Additional pathway enrichment analysis results involving 145 intersecting genes. (A) GO biological processes (BP)-based analysis. (B) GO cellular components (CC) -based analysis.

Supplementary figure S2. Additional pathway enrichment analysis results involving 226 *T. flammea* targets. (A) GO biological processes (BP)-based analysis. (B) GO cellular components (CC) -based analysis.

Supplementary figure S3. Molecular dynamics simulation results of three compounds bound to target proteins (replicate 2)

Supplementary figure S4. Molecular dynamics simulation results of three compounds bound to target proteins (replicate 3)

Supplementary figure S5. Protein–ligand contact profiles for three compounds bound to target proteins.

Supplementary tables (in separate spreadsheet)

Supplementary table S1. Full list of identified metabolites from *T. flammea* extract

Supplementary table S2. Full list of skin aging-related genes identified from Genecards, Open Targets & SenSkin™ databases

Supplementary table S3. Detailed information of PPI network consists of 145 intersecting genes

Supplementary table S4. Full pathway enrichment analysis results involving 145 intersecting genes

Supplementary table S5. Full list of *T. flammea* metabolite-protein association annotations collected from PubChem pre-compiled dataset

Supplementary table S6. Full list of *T. flammea* metabolite-protein association annotations collected from PubChem bioassay dataset

Supplementary table S7. Full list of *T. flammea* metabolite-protein association prediction results produced with SwissTargetPrediction

Supplementary table S8. Full list of *T. flammea* metabolite-protein association prediction results produced with STITCH

Supplementary table S9. Non-redundant list of *T. flammea* metabolite-protein association pairs identified from available databases

Supplementary table S10. Detailed information of PPI network consists of 226 *T. flammea*-associated skin aging targets

Supplementary table S11. Full pathway enrichment analysis results involving 226 *T. flammea* targets

Supplementary table S12. Summary of protein target structures and corresponding PDB IDs used for molecular docking analysis.

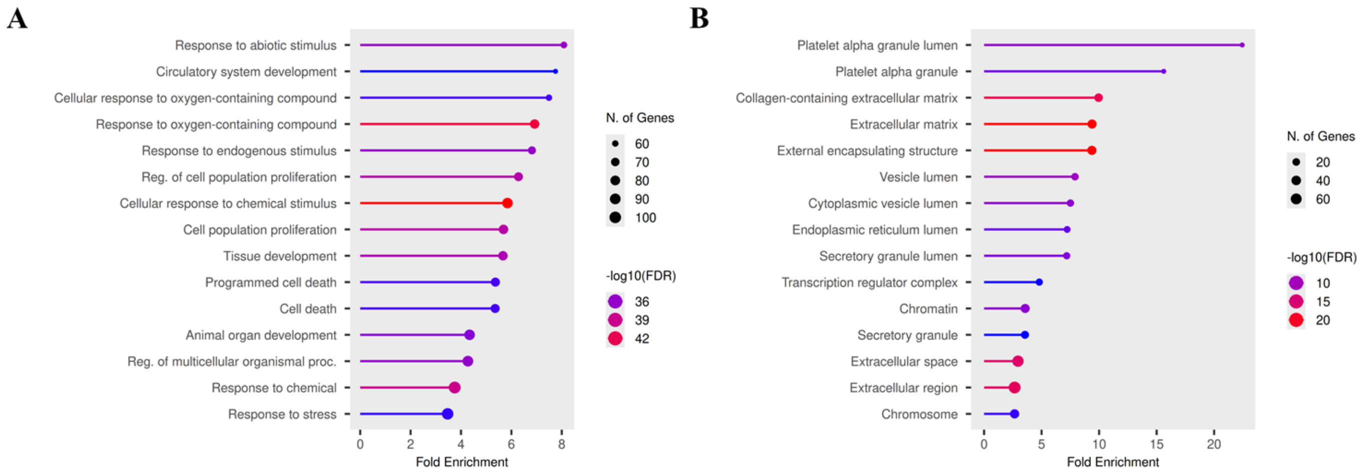

Supplementary figure S1. Additional pathway enrichment analysis results involving 145 intersecting genes. **(A)** GO biological processes (BP)-based analysis. **(B)** GO cellular components (CC) -based analysis.

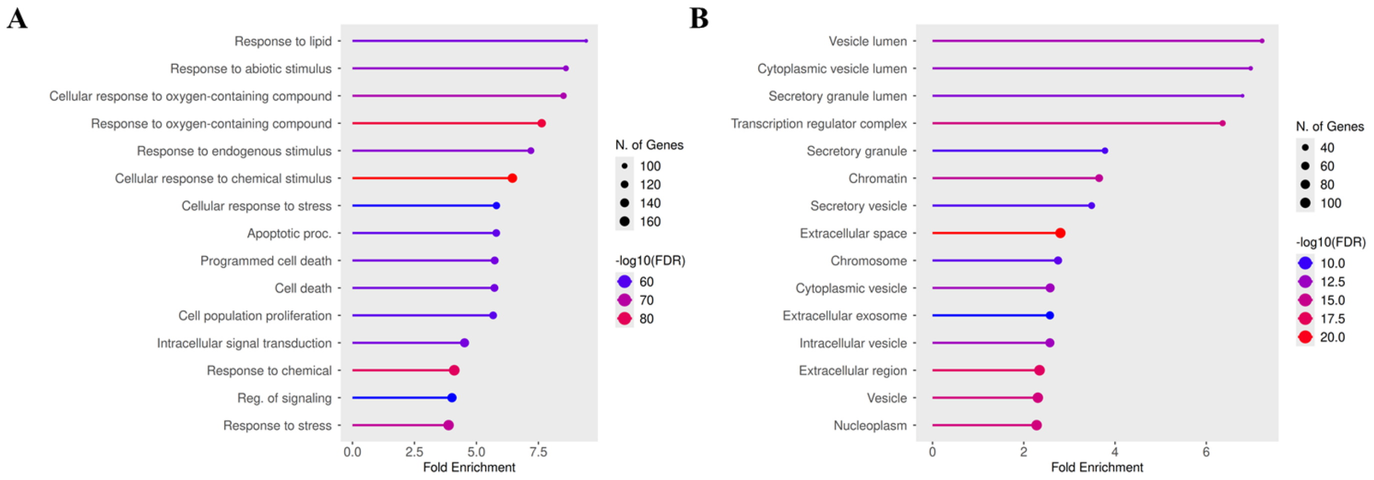

Supplementary figure S2. Additional pathway enrichment analysis results involving *T. flammea* targets. **(A)** GO biological processes (BP)-based analysis. **(B)** GO cellular components (CC) -based analysis.

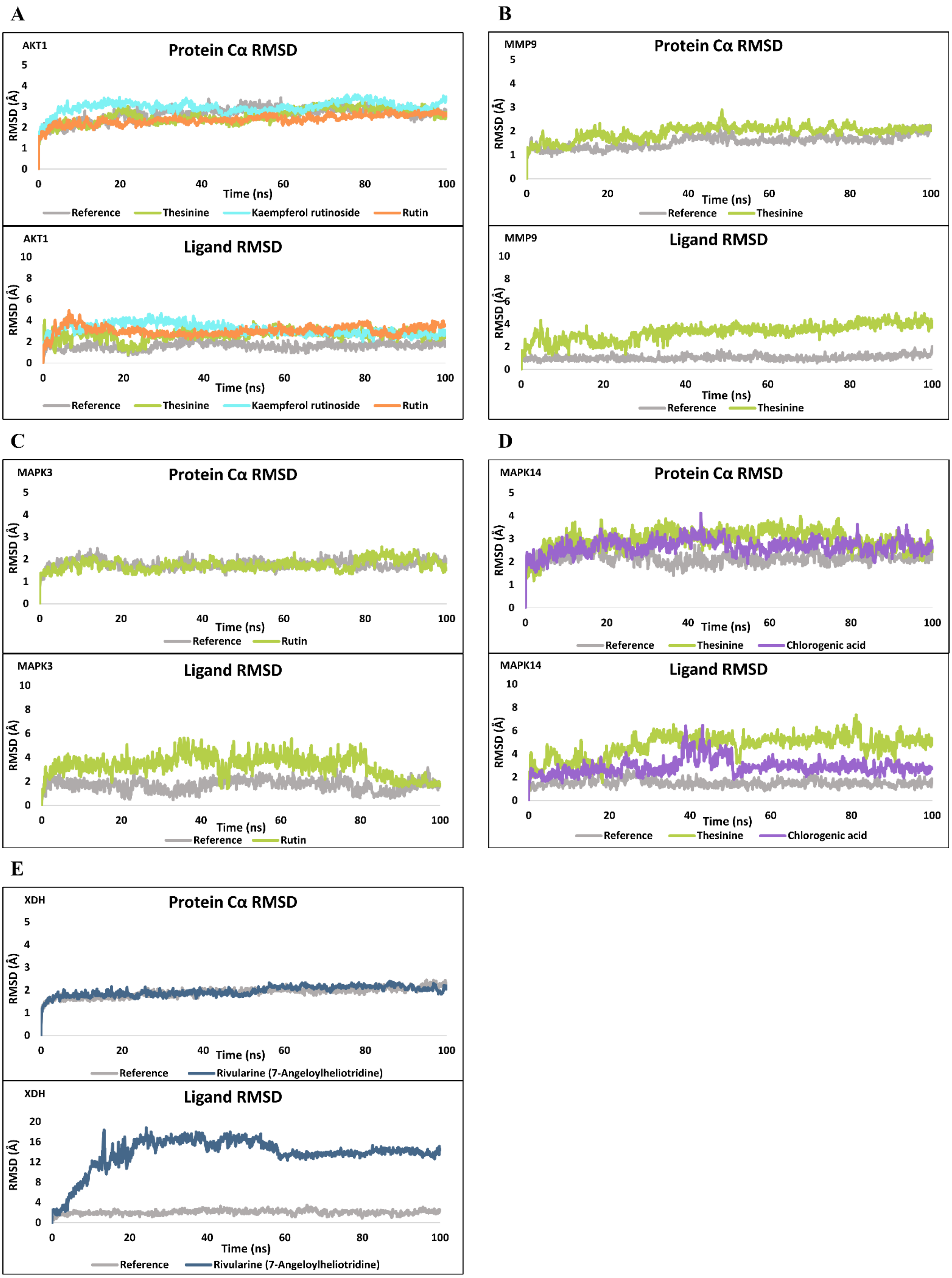
A
AKT1
Protein Cα RMSD
RMSD (Å)
Time (ns)
Reference
Thesinine
Kaempferol rutinoside
Rutin
AKT1
Ligand RMSD
RMSD (Å)
Time (ns)
Reference
Thesinine
Kaempferol rutinoside
Rutin
B
MMP9
Protein Cα RMSD
RMSD (Å)
Time (ns)
Reference
Thesinine
MMP9
Ligand RMSD
RMSD (Å)
Time (ns)
Reference
Thesinine
C
MAPK3
Protein Cα RMSD
RMSD (Å)
Time (ns)
Reference
Rutin
MAPK3
Ligand RMSD
RMSD (Å)
Time (ns)
Reference
Rutin
D
MAPK14
Protein Cα RMSD
RMSD (Å)
Time (ns)
Reference
Thesinine
Chlorogenic acid
MAPK14
Ligand RMSD
RMSD (Å)
Time (ns)
Reference
Thesinine
Chlorogenic acid
E
XDH
Protein Cα RMSD
RMSD (Å)
Time (ns)
Reference
Rivularine (7-Angeloylheliotridine)
XDH
Ligand RMSD
RMSD (Å)
Time (ns)
Reference
Rivularine (7-Angeloylheliotridine)

Supplementary figure S3. Molecular dynamics simulation results of three compounds bound to target proteins (replicate 2). Time-dependent root-mean-square deviation (RMSD) plots of protein Cα atoms and bound ligands during 100 ns MD simulations are shown for each target–ligand complex. The “reference” trace corresponds to the co-crystallized ligand from each protein structure. Thesinine, Rutin, Kaempferol rutinoside, Chlorogenic acid, and Rivularine are depicted as green, orange, cyan, purple, and deep-blue trajectories, respectively. **(A)** AKT1. **(B)** MMP9. **(C)** MAPK3. **(D)** MAPK14. **(E)** XDH.

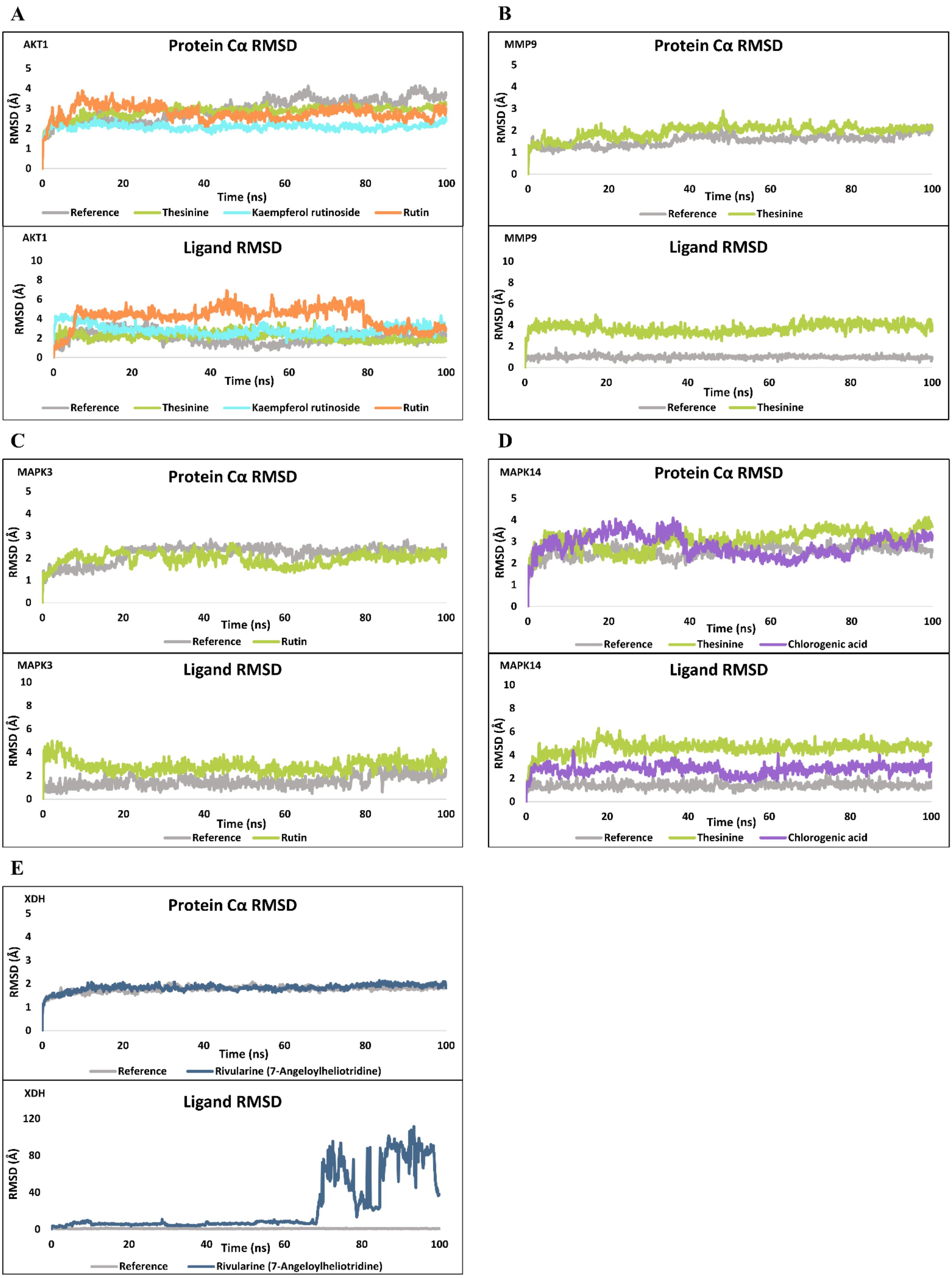
A
AKT1
Protein Cα RMSD
RMSD (Å)
Time (ns)
Reference
Thesinine
Kaempferol rutinoside
Rutin
AKT1
Ligand RMSD
RMSD (Å)
Time (ns)
Reference
Thesinine
Kaempferol rutinoside
Rutin
B
MMP9
Protein Cα RMSD
RMSD (Å)
Time (ns)
Reference
Thesinine
MMP9
Ligand RMSD
RMSD (Å)
Time (ns)
Reference
Thesinine
C
MAPK3
Protein Cα RMSD
RMSD (Å)
Time (ns)
Reference
Rutin
MAPK3
Ligand RMSD
RMSD (Å)
Time (ns)
Reference
Rutin
D
MAPK14
Protein Cα RMSD
RMSD (Å)
Time (ns)
Reference
Thesinine
Chlorogenic acid
MAPK14
Ligand RMSD
RMSD (Å)
Time (ns)
Reference
Thesinine
Chlorogenic acid
E
XDH
Protein Cα RMSD
RMSD (Å)
Time (ns)
Reference
Rivularine (7-Angeloylheliotridine)
XDH
Ligand RMSD
RMSD (Å)
Time (ns)
Reference
Rivularine (7-Angeloylheliotridine)

Supplementary figure S4. Molecular dynamics simulation results of three compounds bound to target proteins (replicate 3). Time-dependent root-mean-square deviation (RMSD) plots of protein Cα atoms and bound ligands during 100 ns MD simulations are shown for each target–ligand complex. The "reference" trace corresponds to the co-crystallized ligand from each protein structure. Thesinine, Rutin, Kaempferol rutinoside, Chlorogenic acid, and Rivularine are depicted as green, orange, cyan, purple, and deep-blue trajectories, respectively. **(A)** AKT1. **(B)** MMP9. **(C)** MAPK3. **(D)** MAPK14. **(E)** XDH.

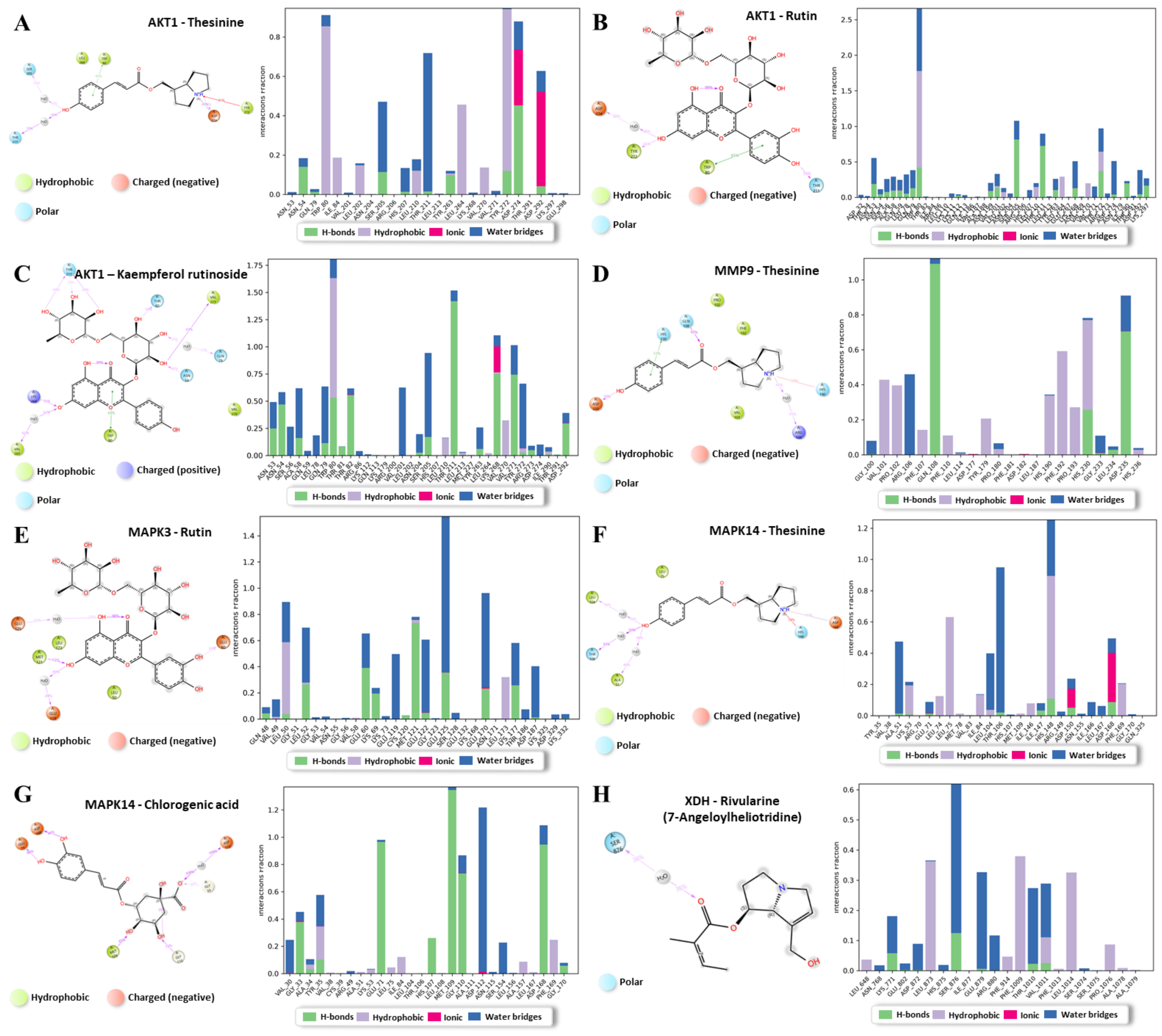

Supplementary figure S5. Protein–ligand contact profiles for three compounds bound to target proteins. The upper panel of each subfigure displays the 2D interaction diagram, summarizing key contacts formed between the ligand and the target protein. Only interactions that persist for more than 30% of the total simulation time are shown. The lower panel illustrates the corresponding protein–ligand interaction fraction histogram throughout the simulation.
